# Survival outcomes of combined hepatocellular‐cholangiocarcinoma compared with intrahepatic cholangiocarcinoma: A SEER population‐based cohort study

**DOI:** 10.1002/cam4.4474

**Published:** 2021-12-04

**Authors:** Zhen Yang, Guangjun Shi

**Affiliations:** ^1^ Department of Hepatopancreatobiliary Surgery Qingdao Municipal Hospital Qingdao University Qingdao P.R. China

## Abstract

**Background:**

Combined hepatocellular‐cholangiocarcinoma (CHC) is a heterogeneous group of primary liver cancers characterized by the coexistence of both hepatic and biliary cellular contents. The aim of this study was to compare CHC and intrahepatic cholangiocarcinoma (ICC) and investigate the treatment and survival of patients with CHC.

**Methods:**

Data on CHC and ICC, including clinicopathological characteristics, treatments, and survival outcomes were extracted from the SEER database between 2004 and 2016. Univariate and multivariate analyses of all data were performed to identify the risk factors associated with survival outcomes. The overall survival (OS) rates of CHC patients who underwent hepatic resection (HR) or liver transplantation (LT) were also assessed before and after propensity score matching.

**Results:**

A total of 1066 consecutive patients who had been diagnosed with CHC (*n* = 286) or ICC (*n* = 780) were identified. The mean age of the CHC cohort was 60.8±10.7 years old. Among the CHC group, a large proportion of the patients were men and of White ethnicity (73.1% and 71.3%, respectively). The majority of tumors were poorly differentiated (37.8%), while the most common AJCC stage at presentation was stage I (31.4%). Multivariable analysis of all CHC patients revealed that only tumor size, M_1_ stage, AJCC stage IIIC, AJCC stage IV, surgery, and chemotherapy were significantly associated with OS. The OS was comparable with the ICC in the initial 36 months and better in the subsequent follow‐up after treatment. Surgery was associated with better survival outcomes, whether in the early or advanced stages. Regarding the specific types of surgery, the OS rates were similar in selected patients following HR or LT.

**Conclusion:**

In patients with CHC, surgical intervention resulted in better long‐term survival outcomes than nonsurgical treatments. The OS rate of CHC patients compared with that of ICC patients was discriminated before and after a 3‐year follow‐up.

## Graphical abstract

In patients with CHC, surgical intervention provided better long‐term survival outcomes in comparison with nonsurgical treatments. The overall survival rate of CHC patients compared with ICC patients discriminated before and after 3‐year follow‐up.

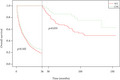

## 1 INTRODUCTION

Combined hepatocellular‐cholangiocarcinoma (CHC) is a rare subtype of primary liver cancer with biphenotypic characteristics of both hepatocytic and cholangiocytic differentiation, accounting for 0.4%–14.2% of primary liver malignancies.^1^, ^2^, ^3^ As the name implies, CHC was currently viewed as a constitution with the dual clinicopathological features of hepatocellular carcinoma (HCC) and intrahepatic cholangiocarcinoma (ICC).^4^, ^5^ Due to its rarity and dual nature, the disease is not yet well understood. The ambiguous biological features of CHC may hinder the formulation of treatment protocols and seriously affect the prognosis of patients. Previous studies have reported that CHC and ICC have comparable survival outcomes.^6^ Molecular analysis suggested that CHC and ICC shared common altered carcinogenic pathways and were genetically closer compared to HCC,^7^ while other genomic profiling uncovered genetic similarities between CHC and HCC.^8^ To date, there have been limited investigations of patients with CHC. As one subtype of the uncommon liver cancers, the demographics, clinical features, survival outcomes, risk factors, and therapeutic landscapes especially the role of radical resection remained largely unknown. Therefore, the purpose of this large population study based on the SEER program was to clearly define the clinicopathologic characteristics and to evaluate the survival outcomes as well as different therapeutic regimens.

## 2 PATIENTS AND METHODS

### 2.1 Database

Patients pathologically diagnosed with CHC and ICC were identified from the Surveillance, Epidemiology, and End Result cancer database (SEER) between 2004 and 2016. A total of 1066 patients were included in this retrospective study. Of these, 286 patients were confirmed to have CHC and 780 patients had ICC. The data on demographics, American Joint Committee on Cancer (AJCC) stages, histopathological characteristics, treatments, and survival outcomes were collected from the database. The patients with incomplete information regarding the clinical variables were not included.

### 2.2 Statistical analysis

Continuous variables were described as means (±SD) or medians (interquartile range) and analyzed using the Student's *t* test or the Mann–Whitney *U* test, as appropriate. Categorical data are shown as number (percentage) and were assessed using the chi‐square (*χ*²) test or Fisher's exact test, as appropriate. The overall survival (OS) and cancer‐specific survival (CSS) curves were calculated using the Kaplan–Meier method and estimated using the log‐rank test. The univariate and multivariate analyses of all data were performed using the Cox proportional hazards regression model. A propensity score matching (PSM) method using logistic regression was utilized to correct the case‐mix in order to overcome the selection biases of baseline covariates between groups.^9^, ^10^ The cohorts were matched for propensity scores at a 1:1 ratio with a caliper of 0.02. The confounding factors included in the model were age, race, tumor size, and marital status. Before PSM, quantitative data were analyzed by the student's *t* test or the Mann–Whitney *U* test, while qualitative data were compared using the *χ* ^2^ test or Fisher's exact probability test, as appropriate. After PSM, the matched groups were compared using a paired *t*‐test or Wilcoxon signed test for continuous variables and the McNemar test for categorical data. A two‐sided *p* value of less than 0.05 was considered as statistically significant. All the analyses were conducted using SPSS version 26.0 and R software version 3.6.2.

## 3 RESULTS

### 3.1 Baseline characteristics

A total of 1066 patients were enrolled in this study between 1975 and 2016. The baseline characteristics of all patients and the comparison of CHC and ICC patients are summarized in Table 1. Among the patients, 286 and 780 patients were in the CHC and ICC cohorts, respectively. The clinicopathological features in Table 1 were significantly different except for the marital status, combined metastasis in the brain, and whether or not the patient received radiation. The CHC group included a larger proportion of men (73.1% vs. 26.9%) and had a majority of White ethnicity (71.3%, 204/286). The mean age of the CHC cohort was 62.8 ± 10.7 years old, which was significantly younger than the ICC cohort (*p* < 0.001). The patients in the CHC group showed a distinctly higher incidence of elevated AFP levels compared to the patients in the ICC group (53.5% vs. 17.7%, *p* < 0.001). Notably, the rate of liver fibrosis was significantly higher in CHC patients (12.2% vs. 3.8%, *p* < 0.001). A large proportion of CHC patients were diagnosed at early stages (stage I and II: 56.6%). Of the patients, 52.8% (151/286) underwent surgery and 42.0% (120/286) underwent chemotherapy in the CHC cohort. Detailed data are presented in Table 1.

**TABLE 1:** Baseline characteristics of CHC and ICC patients

Variables	All patients (*n* = 1066)	CHC (*n* = 286)	ICC (*n* = 780)	*p* value
Gender				**<0.001**
Male	594 (55.7%)	209 (73.1%)	385 (49.4%)	
Female	472 (44.3%)	77 (26.9%)	395 (50.6%)	
Age (years), ±SD	62.8 ± 11.4	60.8 ± 10.7	63.6 ± 11.6	**<0.001**
Race				**<0.001**
Black	100 (9.4%)	29 (10.1%)	71 (9.1%)	
White	835 (78.3%)	204 (71.3%)	631 (80.9%)	
Other	131 (12.3%)	53 (18.5%)	78 (10.0%)	
Tumor size (cm), ±SD	7.02 ± 4.00	6.24 ± 4.32	7.30 ± 3.84	**<0.001**
Marital status				0.608
Married	620 (58.2%)	170 (59.4%)	450 (57.7%)	
Other	446 (41.8%)	116 (40.6%)	330 (42.3%)	
AFP level				**<0.001**
Positive	291 (27.3%)	153 (53.5%)	138 (17.7%)	
Negative	366 (34.3%)	75 (26.3%)	291 (37.3%)	
Borderline	2 (0.2%)	1 (0.3%)	1 (0.1%)	
Unknown	407 (38.2%)	57 (19.9%)	350 (44.9%)	
Fibrosis				**<0.001**
Severe or cirrhosis	65 (6.1%)	35 (12.2%)	30 (3.8%)	
None or unknown	1001 (93.9%)	251 (87.8%)	750 (96.2%)	
Grade				**<0.001**
Well	47 (4.4%)	11 (3.8%)	36 (4.6%)	
Moderately	298 (28.0%)	63 (22.0%)	235 (30.1%)	
Poorly	289 (27.1%)	108 (37.8%)	181 (23.2%)	
Undifferentiated	11 (1.0%)	8 (2.8%)	3 (0.4%)	
Unknown	421 (39.5%)	96 (33.6%)	325 (41.7%)	
T stage				**<0.001**
T1	417 (39.1%)	109 (38.1%)	308 (39.4%)	
T2	202 (18.9%)	89 (31.2%)	113 (14.5%)	
T3	337 (31.6%)	69 (24.1%)	268 (34.4%)	
T4	91 (8.5%)	17 (5.9%)	74 (9.5%)	
TX	19 (1.8%)	2 (0.7%)	17 (2.2%)	
N stage				**0.001**
N0	808 (75.8%)	240 (83.9%)	568 (72.8%)	
N1	214 (20.1%)	39 (13.7%)	175 (22.5%)	
NX	44 (4.1%)	7 (2.4%)	37 (4.7%)	
M stage				**0.013**
M0	783 (73.5%)	226 (79.0%)	557 (71.4%)	
M1	283 (26.5%)	60 (21.0%)	223 (28.6%)	
AJCC stage				**<0.001**
I	307 (28.8%)	90 (31.4%)	217 (27.8%)	
II	144 (13.5%)	72 (25.2%)	72 (9.2%)	
IIIA	162 (15.2%)	34 (11.9%)	128 (16.4%)	
IIIB	41 (3.8%)	10 (3.5%)	31 (4.0%)	
IIIC	129 (12.1%)	20 (7.0%)	109 (14.0%)	
IV	283 (26.6%)	60 (21.0%)	223 (28.6%)	
Combined Mets at brain	1 (0.1%)	0	1 (0.1%)	1.00
Combined Mets at bone	32 (3.0%)	3 (1.0%)	29 (3.7%)	**0.024**
Combined Mets at lung	58 (5.4%)	9 (3.1%)	49 (6.3%)	**0.046**
Surgery				**<0.001**
Done	431 (40.4%)	151 (52.8%)	280 (35.9%)	
None	635 (59.6%)	135 (47.2%)	500 (64.1%)	
Radiation				0.097
Done	53 (5.0%)	9 (3.1%)	44 (5.6%)	
None	1013 (95.0%)	277 (96.9%)	736 (94.4%)	
Chemotherapy				**0.010**
Done	517 (48.5%)	120 (42.0%)	397 (50.9%)	
No/unknown	549 (51.5%)	166 (58.0%)	383 (49.1%)	

Bold values indicate *p* < 0.05.

Abbreviations: AJCC, American joint committee on cancer; CHC, combined hepatocellular‐cholangiocarcinoma; ICC, intrahepatic cholangiocarcinoma; Mets, metastasis; SD, standard deviation.

### 3.2 Survival outcomes

**FIGURE 1:**
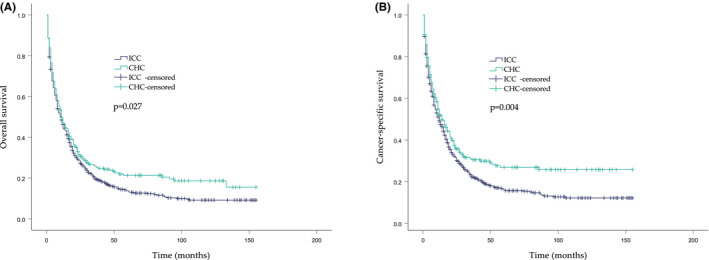
Estimates of survival between CHC and ICC patients. (A) Overall survival rates. (B) Cancer‐specific survival rates

**FIGURE 2:**
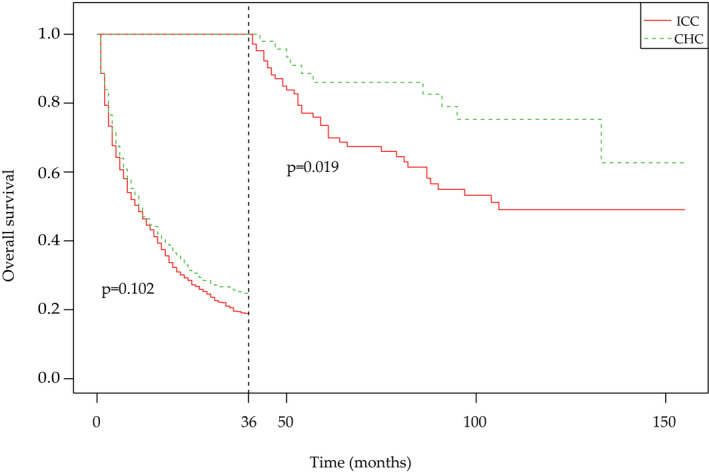
Overall survival curves for the landmark analysis in CHC patients

Of all the enrolled patients, the OS and cancer‐specific survival (CSS) were significantly better in the CHC group than in the ICC group. (Figure 1) The 1‐year, 3‐year, and 5‐year OS probabilities were 47.1%, 25.7%, and 21.3% in the CHC group and 46.4%, 19.6%, and 13.8% in the ICC group, respectively. The 1‐year, 3‐year, and 5‐year CSS probabilities were 52.1%, 30.5%, and 26.9% in the CHC group and 49.2%, 22.3%, and 16.3% in the ICC group, respectively. The 1‐year OS and CSS were comparable between the two groups (*p* > 0.05). Additionally, the 3‐year and 5‐year OS and CSS were significantly lower in the ICC group than in the CHC group (*p* < 0.05). (Table 2) Further landmark analysis of all patients in the first 36 months of treatment showed no significant difference in the OS between the CHC and ICC groups. However, the similarity in the OS of CHC patients compared to ICC patients within 36 months was lost at the subsequent follow‐up. (Figure 2) In other words, an OS rate estimate of less than 3 years might not be sufficient to evaluate the long‐term clinical outcomes in patients with CHC.

**TABLE 2:** Survival outcomes of patients with CHC and ICC

Outcomes	CHC (*n* = 286)	ICC (*n* = 780)	*p* value
Overall survival			
1‐year	47.1%	46.4%	0.421
3‐year	25.7%	19.6%	**0.024**
5‐year	21.3%	13.8%	**<0.001**
Cancer‐specific survival			
1‐year	52.1%	49.2%	0.203
3‐year	30.5%	22.3%	**<0.001**
5‐year	26.9%	16.3%	**<0.001**

Bold values indicate *p* < 0.05.

Abbreviations: CHC, combined hepatocellular‐cholangiocarcinoma; ICC, intrahepatic cholangiocarcinoma.

### 3.3 Overall survival of CHC and ICC patients at different stages

**FIGURE 3:**
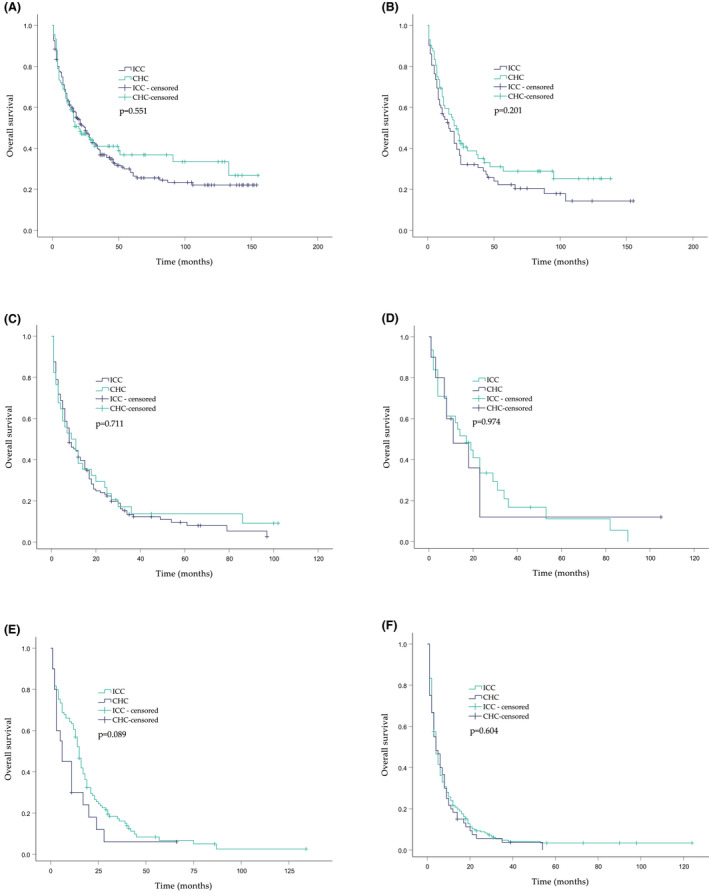
Kaplan–Meier curves of overall survival stratified by the AJCC stages. (A) Stage Ⅰ. (B) Stage Ⅱ. (C) Stage ⅢA. (D) Stage ⅢB. (E) Stage ⅢC. (F) Stage Ⅳ

The OS rates of CHC patients at any stage were not significantly different from those of ICC patients at the corresponding stage (*p* > 0.05). (Figure 3) However, landmark analyses to access the OS probabilities by dividing the entire follow‐up period into the initial 3 years and the subsequent years revealed different patterns.

### 3.4 Treatment patterns and the relative survival outcomes in CHC patients

**FIGURE 4:**
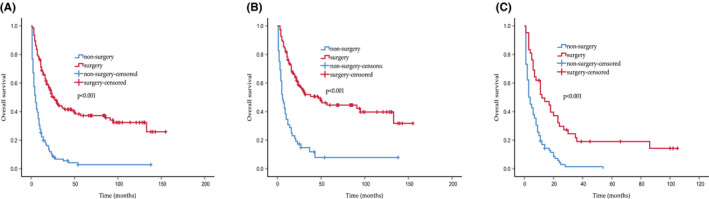
Kaplan–Meier curves of overall survival in CHC patients with surgery and non‐surgery treatment. (A) The entire patients. (B) The early stages patients. (C) The advanced stages patients

The treatment patterns of the different stages are summarized in Table 3. Surgery and chemotherapy remained the main treatment options, whereas radiation was rarely performed in the CHC cohort. The majority of early stage patients (stage I and II) underwent surgery and were associated with a better OS than other treatment options in the CHC group (*p* < 0.001). As for the advanced stages (stages III and IV), surgery also demonstrated more favorable OS outcomes compared with nonsurgical modalities (*p* < 0.001, Figure 4).

**TABLE 3:** Treatment patterns of CHC patients in different AJCC stages

Patterns	AJCC stages						
	Ⅰ	Ⅱ	ⅢA	ⅢB	ⅢC	Ⅳ	Total
Surgery only	51	33	8	3	4	3	102
Chemotherapy only	8	13	9	3	6	32	71
Radiation only	0	0	0	0	0	1	1
Surgery‐chemotherapy	9	16	8	2	7	6	48
Trimodality	1	0	1	0	0	4	6
None of the three	21	10	9	2	3	17	62

Abbreviations: AJCC, American joint committee on cancer; CHC, combined hepatocellular‐cholangiocarcinoma.

### 3.5 Overall survival of CHC patients with different surgical types

**FIGURE 5:**
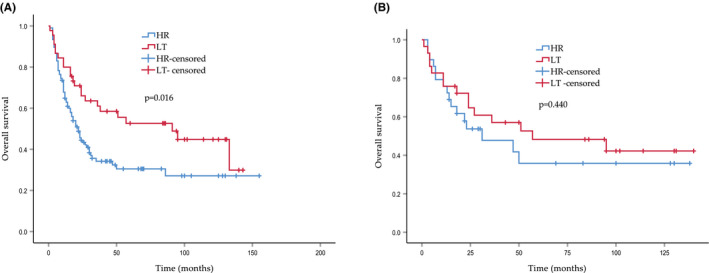
Kaplan–Meier curves of overall survival in CHC patients with hepatic resection (HR) and liver transplantation (LT). (A) Survival curves in unmatched patients. (B) Survival curves in matched patients

Of all the patients in the CHC group, 106 patients underwent hepatic resection (HR) and 45 patients underwent liver transplantation (LT). Notably, patients who underwent HR tended to be older (60.58 ± 9.62 vs. 55.87 ± 9.71, *p* = 0.007) and had a larger tumor size (6.14 ± 3.66 cm vs. 3.12 ± 2.07 cm, *p* < 0.001). In addition, marital status and race differed significantly between the two groups. Except for these remarkably different characteristics, the remaining baseline demographic data did not show statistical differences (Table 4); after propensity score matching (PSM), 29 patients were identified in each matched cohort, and the baseline parameters were well balanced. (Table 4) The long‐term outcomes were compared between the HR and LT groups by evaluating the OS before and after PSM. The OS probabilities were significantly different between the two unmatched cohorts (*p* = 0.016), while in the matched group, the OS rates were comparable regardless of the surgical type (*p* = 0.440, Figure 5).

**TABLE 4:** Demographic and clinical characteristics of patients undergoing HR or LT before and after PSM

Variables	Before PSM			After PSM		
	HR (*n* = 106)	LT (*n* = 45)	*p* value	HR (*n* = 29)	LT (*n* = 29)	*p* value
Gender			0.375			1.00
Male	75 (70.8%)	35 (77.8%)		21 (72.4%)	22 (75.9%)	
Female	31 (29.2%)	10 (22.2%)		8 (27.6%)	7 (24.1%)	
Age (years), ±SD	60.58 ± 9.62	55.87 ± 9.71	**0.007**	60.52 ± 10.61	58.41 ± 6.86	0.245
Race			**0.033**			0.343
Black	7 (6.6%)	5 (11.1%)		3 (10.3%)	1 (3.4%)	
White	73 (68.9%)	37 (82.2%)		20 (69.0%)	25 (86.3%)	
Other	26 (24.5%)	3 (6.7%)		6 (20.7%)	3 (10.3%)	
Tumor size (cm), ±SD	6.14 ± 3.66	3.12 ± 2.07	**<0.001**	3.78 ± 2.71	3.53 ± 2.31	0.467
Marital status			**0.014**			1.00
Married	72 (67.9%)	21 (46.7%)		12 (41.4%)	11 (37.9%)	
Other	34 (32.1%)	24 (53.3%)		17 (58.6%)	18 (62.1%)	
AFP level			0.592			0.684
Positive	58 (54.8%)	20 (44.5%)		13 (44.8%)	15 (51.7%)	
Negative	26 (24.5%)	14 (31.1%)		10 (34.5%)	7 (24.1%)	
Borderline	1 (0.9%)	0		1 (3.4%)	0	
Unknown	21 (19.8%)	11 (24.4%)		5 (17.2%)	7 (24.1%)	
Fibrosis			0.722			0.238
Severe or cirrhosis	14 (13.2%)	5 (11.1%)		7 (24.1%)	4 (13.8%)	
None or unknown	92 (86.8%)	40 (88.9%)		22 (75.9%)	25 (86.2%)	
Grade			0.402			0.503
Well	3 (2.8%)	2 (4.4%)		0	1 (3.4%)	
Moderately	28 (26.4%)	17 (37.8%)		7 (24.1%)	11 (34.0%)	
Poorly	46 (43.5%)	13 (28.9%)		10 (34.5%)	10 (34.5%)	
Undifferentiated	5 (4.7%)	1 (2.2%)		2 (6.9%)	1 (3.4%)	
Unknown	24 (22.6%)	12 (26.7%)		10 (34.5%)	6 (20.7%)	
T stage			0.182			0.060
T1	46 (43.4%)	17 (37.8%)		17 (58.7%)	7 (24.1%)	
T2	34 (32.1%)	22 (48.9%)		9 (31.0%)	16 (55.3%)	
T3	18 (17.0%)	5 (11.1%)		2 (6.9%)	5 (17.2%)	
T4	8 (7.5%)	1 (2.2%)		1 (3.4%)	1 (3.4%)	
N stage			0.579			0.237
N0	96 (90.6%)	42 (93.3%)		29 (100%)	26 (89.7%)	
N1	10 (9.4%)	3 (6.7%)		0	3 (10.3%)	
M stage			0.608			1.00
M0	99 (93.4%)	43 (95.6%)		28 (96.6%)	27 (93.1%)	
M1	7 (6.6%)	2 (4.4%)		1 (3.4%)	2 (6.9%)	
AJCC stage			0.405			0.450
I	44 (41.5%)	17 (37.8%)		17 (58.7%)	7 (24.1%)	
II	30 (28.3%)	19 (42.3%)		8 (27.6%)	13 (44.9%)	
IIIA	11 (10.4%)	5 (11.1%)		2 (6.9%)	5 (17.2%)	
IIIB	5 (4.7%)	0		1 (3.4%)	0	
IIIC	9 (8.5%)	2 (4.4%)		0	2 (6.9%)	
IV	7 (6.6%)	2 (4.4%)		1 (3.4%)	2 (6.9%)	

Bold values indicate *p* < 0.05.

Abbreviations: AJCC, American joint committee on cancer; HR, hepatic resection; LT, liver transplantation; PSM, propensity score matching; SD, standard deviation.

### 3.6 Analysis of prognostic factors for survival outcomes

Univariate analysis of all CHC patients revealed that the age at diagnosis, tumor size, T stage (T_3_, T*_x_*), N stage (N_1_, N*_X_*), M_1_ stage, advanced AJCC stage (IIIC, IV), lung metastasis, surgical intervention, and chemotherapy were the risk factors for OS. On multivariate Cox regression analysis, only the tumor size (hazard ratio [HR] 1.01; 95% CI 1.00–1.01; *p *= 0.020), M_1_ stage (HR, 1.63; 95% CI, 1.04–2.56; *p* = 0.033), AJCC stage IIIC (HR 2.27; 95% CI 1.22–4.24, *p *= 0.010), AJCC stage IV (HR 2.41; 95% CI 1.47–3.97, *p* = 0.001), surgery (HR 0.29; 95% CI 0.20–0.43, *p* < 0.001), and chemotherapy (HR 0.64; 95% CI 0.44–0.94; *p* = 0.024) were significantly associated with the OS. With respect to cancer‐specific survival (CSS), a multivariate analysis identified that the tumor size (HR 1.01; 95% CI 1.00–1.01; *p* = 0.022), T_4_ (HR 2.34; 95% CI 1.15–4.74, *p* = 0.019), M_1_ (HR 1.91; 95% CI 1.20–3.03, *p* = 0.006), AJCC stage IV (HR 2.64; 95% CI 1.56–4.47, *p* < 0.001), and surgical intervention (HR 0.29; 95% CI 0.19–0.43, *p* < 0.001) were the independent predictors of CSS. (Table 5).

**TABLE 5:** Univariate and multivariate analyses for overall survival and cancer‐specific survival in CHC patients

Variables	Overall survival				Cancer‐specific survival			
	Univariate analysis		Multivariate analysis		Univariate analysis		Multivariate analysis	
	HR (95% CI)	*p* value	HR (95% CI)	*p* value	HR (95% CI)	*p* value	HR (95% CI)	*p* value
Age	1.02 (1.00–1.03)	**0.027**	1.00 (0.99–1.02)	0.885	1.02 (1.00–1.03)	**0.022**	1.01 (0.99–1.02)	0.411
Sex								
Female	Ref				Ref			
Male	1.20 (0.86–1.67)	0.296			1.14 (0.80–1.63)	0.480		
Race								
Black	Ref				Ref			
White	0.86 (0.53–1.40)	0.553			0.97 (0.56–1.65)	0.899		
Other	0.80 (0.46–1.41)	0.444			0.88 (0.47–1.65)	0.690		
Tumor size	1.01 (1.00–1.01)	**<0.001**	1.01 (1.00–1.01)	**0.020**	1.01 (1.00–1.01)	**<0.001**	1.01 (1.00–1.01)	**0.022**
Martial status								
Married	Ref				Ref			
Other	1.20 (0.90–1.61)	0.210			1.15 (0.84–1.58)	0.380		
AFP level								
Negative	Ref				Ref			
Borderline	0.48 (0.06–3.24)	0.426			0.01 (0.01‐4E+154)	0.953		
Positive	0.90 (0.64–1.26)	0.532			0.83 (0.58–1.19)	0.316		
Unknown	0.81 (0.53–1.25)	0.340			0.85 (0.54–1.33)	0.469		
Fibrosis								
None or unknown	Ref				Ref			
Severe or cirrhosis	1.04 (0.66–1.62)	0.877			1.09 (0.68–1.74)	0.718		
Grade								
Well	Ref				Ref			
Moderately	0.66 (0.29–1.49)	0.312			0.57 (0.25–1.31)	0.183		
Poorly	1.48 (0.68–3.22)	0.318			1.26 (0.58–2.74)	0.565		
Undifferentiated	0.92 (0.29–2.90)	0.887			0.72 (0.21–2.47)	0.603		
Unknown	1.12 (0.51–2.44)	0.779			0.95 (0.43–2.09)	0.896		
T stage								
T1	Ref		Ref		Ref		Ref	
T2	1.14 (0.81–1.61)	0.451	1.36 (0.95–1.94)	0.093	1.14 (0.77–1.67)	0.516	1.33 (0.90–1.98)	0.157
T3	1.95 (1.33–2.84)	**0.001**	1.43 (0.91–2.24)	0.122	2.26 (1.51–3.38)	**<0.001**	1.58 (0.98–2.54)	0.063
T4	1.76 (0.93–3.35)	0.083	1.88 (0.94–3.76)	0.072	2.15 (1.12–4.13)	**0.021**	2.34 (1.15–4.74)	**0.019**
TX	18.0 (2.38–136.4)	**0.005**	4.48 (0.44–45.94)	0.207	23.0 (3.00–176.65)	**0.003**	6.56 (0.59–72.43)	0.125
N stage								
N0	Ref		Ref		Ref		Ref	
N1	2.02 (1.35–3.04)	**0.001**	1.19 (0.74–1.92)	0.462	1.97 (1.27–3.06)	**0.002**	0.97 (0.59–1.62)	0.912
NX	4.55 (1.84–11.25)	**0.001**	1.36 (0.46–4.06)	0.577	4.14 (1.51–11.35)	**0.006**	0.91 (0.26–3.11)	0.875
M stage								
M0	Ref		Ref		Ref		Ref	
M1	2.74 (1.92–3.91)	**<0.001**	1.63 (1.04–2.56)	**0.033**	3.18 (2.20–4.59)	**<0.001**	1.91 (1.20–3.03)	**0.006**
AJCC stage								
I	Ref		Ref		Ref		Ref	
II	1.08 (0.73–1.59)	0.703	1.25 (0.84–1.87)	0.271	1.05 (0.69–1.63)	0.802	1.21 (0.77–1.89)	0.402
IIIA	1.58 (0.96–2.62)	0.074	1.54 (0.89–2.69)	0.126	1.84 (1.08–3.12)	**0.024**	1.65 (0.92–2.96)	0.093
IIIB	1.51 (0.65–3.50)	0.343	1.41 (0.58–3.45)	0.447	1.85 (0.79–4.34)	0.159	1.72 (0.69–4.24)	0.242
IIIC	2.24 (1.26–3.98)	**0.006**	2.27 (1.22–4.24)	**0.010**	1.98 (1.02–3.84)	**0.044**	1.87 (0.92–3.82)	0.084
IV	3.25 (2.15–4.92)	**<0.001**	2.41 (1.47–3.97)	**0.001**	3.83 (2.47–5.92)	**<0.001**	2.64 (1.56–4.47)	**<0.001**
Combined Mets at brain	NA	NA			NA	NA		
Combined Mets at bone	2.42 (0.77–7.63)	0.132			2.74 (0.87–8.67)	0.086		
Combined Mets at lung	4.24 (1.97–9.14)	**<0.001**	1.57 (0.63–3.92)	0.334	4.92 (2.27–10.65)	**<0.001**	1.64 (0.66–4.05)	0.287
Surgery								
None	Ref		Ref		Ref		Ref	
Done	0.30 (0.22–0.40)	**<0.001**	0.29 (0.20–0.43)	**<0.001**	0.27 (0.20–0.38)	**<0.001**	0.29 (0.19–0.43)	**<0.001**
Radiation								
None	Ref				Ref			
Done	1.02 (0.45–2.29)	0.970			1.19 (0.53–2.69)	0.680		
Chemotherapy								
No/unknown	Ref		Ref		Ref		Ref	
Done	0.65 (0.49–0.87)	**<0.001**	0.64 (0.44–0.94)	**0.024**	0.58 (0.42–0.79)	**0.001**	0.66 (0.43–1.02)	0.063

Bold values indicate *p* < 0.05.

Abbreviations: AJCC, American joint committee on cancer; CHC, combined hepatocellular‐cholangiocarcinoma; CI, confidence interval; HR, hazard ratio; Mets, metastasis; NA, not applicable; Ref, reference.

## 4 DISCUSSION

The current study showed that CHC was associated with better long‐term survival outcomes compared with ICC. Surgery and chemotherapy play an increasingly vital role in prolonging the survival. Although the OS rates were comparable at any stage between patients with CHC and ICC, the total OS rates were better in the CHC group. One explanation may be that a large proportion of CHC patients were diagnosed at an early stage.

Containing unequivocal components of both biliary and hepatocellular differentiation, CHC, first reported in 1903, was accompanied by growing clinical concerns due to its distinct clinicopathological features. The latest 2019 WHO classification streamlined its previous histological classification system; additionally, the new edition emphasized CHC simply as a mixed tumor with intimate intermingling elements of HCC and ICC.^11^ Notably, the proportion of these two different components was not immutably fixed and invariable, and might be correlated with radiographic as well as physiological features.^12^, ^13^, ^14^ As a result, the diverse histological characteristics have led to difficulties in accurate diagnosis and posed challenges in therapy.

Till date, the histogenesis of CHC remains controversial, although some related theories have been proposed.^15^, ^16^ Recent evidence has shown that hepatic progenitor cells (HPCs), which reside in the biliary ductules, have the potential to differentiate into hepatic or biliary cells.^17^ Thus, several investigators have speculated that CHC may be derived from HPCs.^13^, ^18^, ^19^

The American Joint Committee on Cancer (AJCC) 8th edition of TNM staging classified CHC and ICC into one category and shared the ICC‐specific staging system.^20^ The AJCC staging systems have been revised several times; however, until recently, the CHC did not own specific protocol. Tian et al.^21^ constructed a risk prediction model based on the clinical parameters to preoperatively discriminate CHC from HCC or ICC and to tailor the optimal treatment.

Are the clinical and pathological features of patients with CHC identical to those of patients with ICC? The answer in the current large population‐based study was “No.” Unlike some previous studies, the baseline characteristics regarding the sex ratio, age at diagnosis, tumor grade and stage, and distant metastasis were significantly different between the two groups. Notably, CHC patients were associated with a high incidence of underlying liver cirrhosis and elevated AFP level in comparison with ICC patients, which was in accordance with previous findings.^22^ However, subsequent studies found that the background of liver cirrhosis was not sufficient and was a prerequisite condition for the occurrence of CHC.^23^ Due to the overlapping imaging features of both HCC and ICC, a misdiagnosis often occurs in CHC patients.^24^, ^25^ Li et al.^26^ proposed that combining elevation or discordance of the AFP level with incompatible imaging features of HCC may lead to a diagnosis of CHC. The distinction between CHC and HCC or ICC may largely depend on the composition ratio.

Surgical resection remains the cornerstone of curative treatment for patients with CHC. In the current study, surgery yielded better survival benefits than any other treatment option in the early stages. As for patients at advanced stages, curative resection was still associated with favorable outcomes compared with non‐surgery. However, both CHC and ICC are regarded as more aggressive malignancies with a worse prognosis than HCC. The 3‐year and 5‐year OS rates were relatively lower than those of HCC reported in previous findings.^27^, ^28^, ^29^ Although there was no significant between‐group difference in the OS rate, the patients in the ICC group, as compared with those in the CHC group, exhibited a significant reduction in the OS rate after the initial 36 months following treatments. Considering the regional lymphadenopathy features caused by the biliary cellular component, aggressive surgical intervention, including major hepatectomy and lymphadenectomy, may improve the poor prognosis of CHC patients.^12^ The role of lymph node dissection in survival benefits remains a matter of disscussion.^30^ In our study, a multivariate analysis in the CHC group revealed that the lymph node status was not an independent risk factor for the OS or CSS. Similarly, the survival benefit of LT remains controversial. It is generally believed that transplantation for CHC patients conferred comparable survival benefits to hepatectomy, albeit inferior to HCC, although there are limited data to substantiate the validity.^31^ Using a propensity score matching analysis, our study revealed that the long‐term outcomes of selected patients with CHC were comparable following HR or LT, which was consistent with previous findings.^32^, ^33^ The standard scheme for systemic chemotherapy remains unclear. CHC patients were more frequently treated according to the treatment strategies for HCC or ICC.^34^, ^35^ Further investigation is warranted to establish a standard regimen for CHC.

Our study had several limitations that should be acknowledged. First, inherent selection biases were unavoidable owing to the retrospective design. Second, the absence of detailed information, such as underlying liver diseases, tumor markers, pathologic features, and progression‐free survival in the SEER database hampered the execution of more specific analyses. Finally, the diagnostic criteria for CHC may be inconsistent because of the varied pathological classifications.

Despite these limitations, our data demonstrated that CHC, as a rare malignancy of the liver, was associated with a similar OS in the initial 36 months, while a better survival in the subsequent follow‐up compared with ICC. Furthermore, surgical intervention could significantly improve the prognosis in the early stages. For advanced stages, surgery may be the optimal option.

## CONFLICT OF INTEREST

No conflict of interest was declared by the authors.

## AUTHOR CONTRIBUTION

Guangjun Shi contributed to the conception and collected data. Zhen Yang designed the study and wrote the manuscript.

## ETHICAL STATEMENT

Ethical approval was not sought from institutional review board (IRB) because of the de‐identified data and the nature of the present study. And on account of the retrospective study design, the requirements for informed consent were waived off.

## Data Availability

The raw data supporting the present study will be available from the corresponding author upon reasonable request.
